# “Why is this relevant for me?”: increasing content relevance enhances student motivation and vitality

**DOI:** 10.3389/fpsyg.2023.1184804

**Published:** 2023-09-28

**Authors:** Marius Ole Johansen, Sigrunn Eliassen, Lucas Matias Jeno

**Affiliations:** ^1^bioCEED – Centre of Excellence in Biology Education, Department of Biology, University of Bergen, Bergen, Norway; ^2^Department of Education, University of Bergen, Bergen, Norway

**Keywords:** self-determination theory, autonomous motivation, vitality, autonomy support, intrinsic motivation, emotional affect, stem

## Abstract

The notion that motivation is imperative for students’ psychological well-being and academic functioning is central to Self-Determination Theory (SDT). According to SDT, different types of motivations can co-occur to a various degree with separate outcomes, depending on the extent of experienced degree of autonomy. In the current study, we investigate how making a learning exercise more relevant for higher education STEM students can affect aspects of student functioning mediated through motivation. In a randomized experiment, results indicate that the students who received a more “relevant” assignment (experimental group) experienced more autonomous forms of motivation relative to the students who received a “generic” or “traditional” exercise (control group). Further, the experimental group reported higher levels of vitality and effort relative to the control group. Using a pre- and post-test design measuring changes in emotional affect during the learning activity, we found that the control group reported an increase in negative affect and a decrease in positive affect. Finally, path analysis showed significant relationships between the type of assignment provided and motivation and student functioning.

## Introduction

Students facing learning activities they find boring and uninteresting can be left with feelings like “Why is this relevant for me?” or “What is the point of learning this?” (Frymier and Shulman, 1995). This disassociation of the perceived importance of learning activities can negatively affect student motivation (Hidi and Harackiewicz, 2000). Motivation is an important constituent in any successful educational contexts as a plethora of studies relates student motivation to positive outcomes such as adaptability, the ability to cope with negative emotions, academic achievements, psychological well-being, engagement, deeper learning, and academic thrive (see, e.g., Bye et al., 2007; Milyavskaya and Koestner, 2010; Núñez and León, 2016; Ryan and Deci, 2017; Cheon et al., 2018). But what happens if teachers encounter students showing little to no interest in learning activities? Should these students be incentivized to learn by external contingencies without experiencing an intrinsic drive to embrace the curriculum? In these situations, research shows that instructors and teachers can accentuate relevance to motivate and engage students in the classroom (see, e.g., Brophy, 1999; Yeager et al., 2014; Canning and Harackiewicz, 2015; Vansteenkiste et al., 2018). In this study, we apply Self-Determination Theory (SDT; Ryan and Deci, 2017) to investigate how making an assignment more relevant for higher education STEM students in a statistics course can impact motivation and aspects of student functioning in a classroom experiment.

### Self-determination theory and motivation

The SDT framework is a meta-theory on human motivation and behavior where the locus of an action or experience varies according to the level of autonomy (Vansteenkiste et al., 2010; Ryan and Deci, 2017). Traditionally, motivation has been differentiated between intrinsic and extrinsic motivation. Individuals experience *intrinsic motivation* whenever the reason for their behavior or action is inherent to the learning activity itself, i.e., they find the activity to be joyful and pleasant (Ryan et al., 2021). Intrinsically motivated students will naturally gravitate toward the learning content out of innate curiosity and elation, and due to its volitional nature, it is considered the ultimate autonomous motivation as students willingly embrace the educational context (Ryan and Deci, 2017; Vansteenkiste et al., 2018). Studies show that intrinsic motivation plays a pivotal role in education as it relates to academic achievements, positive emotions and attitudes, and psychological well-being (Levesque et al., 2004; Taylor et al., 2014).

In contrast to other theories encompassing human motivation, SDT differentiates between types of *extrinsic motivation* depending on the degree of perceived autonomy (Deci and Ryan, 2008). When students are extrinsically motivated, the learning activity becomes a means to achieve outcomes that are disconnected from the learning activity (Vansteenkiste et al., 2010). Since the underlying reasons to attain these outcomes have varying degrees of internalization, extrinsic motivation cannot be characterized by a homogenous construct. Instead, it encompasses a spectrum, where some forms of motivation can be controlling in nature while others more autonomous (Ryan and Deci, 2017).

When students are *externally regulated*, the content of the activity itself is unappealing, and they seek only to meet the expectations and demands set by their peers and teachers to avoid punishment or obtain external approval (Pelletier et al., 2001; Cerasoli et al., 2014). As the activity is performed due to external pressure, it is considered a controlled form of motivation.

Students can also engage in learning activities due to feelings of guilt or shame. When students are *introjected regulated*, the reason for engaging in the learning activity no longer originates from an external contingency, but the behavior is still only partially accepted (Reeve et al., 2002). Although the behavior originates from within the individual, this form of motivation is still considered controlling as the behavior is caused by an internal pressure (Vansteenkiste et al., 2018).

When students can identify or recognize that elements of the learning content hold either long-term or short-term significance, they are *identified regulated* (Vansteenkiste et al., 2010; Jeno et al., 2019; Waterschoot et al., 2019). As the reason for engaging in the activity is now more autonomous, identified regulation is considered the first form of autonomous motivation.

Students engaging in learning activities they not only consider meaningful for their later endeavors but that is also aligned with their personal interests and values are *integrated regulated* (Vansteenkiste et al., 2018). Learning activities are now accompanied by a high degree of self-endorsement since the students recognize the content as an extension of their own skills, abilities, and identities. Due to the volitional nature of integrated regulation, it is considered a higher form of autonomous motivation as behaviors are more self-endorsed, allowing students to act truer to themselves (Vansteenkiste et al., 2018). These autonomous forms of motivations are a stark contrast to *amotivation*, i.e., when a student completely lacks any interest or fail to recognize any value in an activity (Kowal and Fortier, 1999).

Research shows that more autonomous forms of motivation, compared to controlled forms of motivation and amotivation, are positively related to achievements, creativity, and psychological well-being (de Jesus et al., 2013; Núñez and León, 2016). When students are autonomously motivated, they explore and engage with the educational context, where experiences and learning now derives from a principal interest of the subject (Vansteenkiste et al., 2018). In contrast, when students experience controlled motivation, they are less persistent in their tasks, and academic achievements and well-being decreases (Howard et al., 2021).

### Correlates of intrinsic motivation and motivational regulations

When self-endorsed, students are more likely to experience feelings of aliveness with a surplus of energy, a trait that has been linked to effort and persistence in educational settings (Mouratidis et al., 2011). In general, correlations of outcomes such as achievements, effort, and vitality become increasingly more positive as the correlates move along the spectrum from extrinsic regulation to intrinsic motivation (Ryan and Connell, 1989). In contrast, external regulation is associated with sub-optimal outcomes such as psychological ill-being and negative emotions (Pelletier et al., 2001; Van der Kaap-Deeder et al., 2016). Although behavior originates from within the individual when introjected regulated, studies have shown that albeit introjected regulated behaviors tend to be correlated with engagement, persistence, and effort, it is also associated with anxiety and negative affect (Ryan and Connell, 1989; Roth et al., 2009; Skinner et al., 2009). This is due to the conflicting duality of introjection; Someone can still perceive an activity as important while it stands without personal meaning. For instance, a student can be “pushed” to enact (such as doing homework) by an internal pressure in the form of guilt, encouraging behaviors at the cost of experiencing negative emotions and energy depletion (Vansteenkiste et al., 2018). Both integrated and identified regulated will recognize an activity as important and accept the behavior, hence both are connected to a greater sense of volition. Consequently, studies have shown they both positively correlate to both positive affect and effort (Pelletier et al., 2001; Roth et al., 2009). Alongside positive outcomes such as willingness to seek out challenges and increased creativity, autonomous motivation has also been shown to affect vitality, positive emotions, and effort (Burton et al., 2006).

### Fostering internalization, meaningful relevance, and motivation

*Internalization* is the process where an individual turns some external demand into a self-endorsed regulation (Ryan and Deci, 2000a). For a student to internalize learning content, it is imperative that the value of the learning activity is clarified. Although teachers and instructors can explain why the learning activities are meaningful to students, these reassurances may still not be enough for students to identify or perceive the content as important. For instance, research shows that connecting learning content to students’ daily lives is better than providing some arbitrary rationale that the content will be important later in their future endeavors (Assor et al., 2002; Canning and Harackiewicz, 2015). However, if the provided rationale is to be personally endorsed by the students, teachers and instructors must incorporate the students’ reference frames so that the content is meaningful from the students’ perspectives (Canning and Harackiewicz, 2015). That is, the provided rationale will only be perceived as relevant if the learning activity is connected to their own personal beliefs, interests, and values.

Educational contexts that clarify why learning material is relevant involve learning activities that help students perceive the learning material to the realization of the students’ own personal values, beliefs, and interests (Rosenzweig et al., 2020). A relevance-clarifying educational context is then perceived as autonomy supportive since the realization of understanding the learning content is now attuned to the students’ personal interests, thus they are acting true to themselves and hence feel more autonomous during the learning activity (Hulleman et al., 2010). Following a classic example from Assor et al. (2002), a student might not enjoy learning mathematics as it inherently is experienced as abstract and non-tangible. However, the student might experience feelings of autonomy if the student understands that advanced mathematical functions or statistical techniques can greatly enhance their ability to solve complex real-life problems later in their career. STEM students who must take numerical courses as part of their degree but generally are not going to pursue a mathematical discipline (such as biology and earth science students) are very prone to being unable to relate course content to personal experiences and values as it may seem too disconnected from daily life (Everingham et al., 2017). Alongside literacy being an integrative part of work and life in general, numeracy is also considered an essential skill by governments (Dion, 2014), and research have shown that higher education students who achieve well in numeracy courses are more capable of conceptualizing quantitative problem-solving (see e.g., Kuo et al., 2013). Further, mathematical achievements have been shown to predict academic success at the higher education level (Taub et al., 2014). Although the emphasis on fostering relevance is considered an important constituent in experiencing self-determined behavior, most learning activities are not intrinsically motivated (Ryan and Deci, 2000b). However, SDT assumes that in an autonomy supportive context, learning activities having an extrinsic origin can be internalized and therefore experienced as autonomous (Assor et al., 2002), i.e., making learning content more relevant and less abstract can increase experiences of autonomy.

Helping students see value in learning content by providing meaningful assignments has been demonstrated to increase motivation in higher education (Wagner et al., 2006; Hulleman et al., 2010; Brown et al., 2015). In particular, the use of relevance interventions has proven to be quite successful in enhancing motivation, academic achievements, and performance among students (Rosenzweig and Wigfield, 2016). Meaningful relevance can be defined as the perceived importance of an activity due to its usefulness for other tasks or assignments in a person’s life or goals (Eccles et al., 1983). A student is more likely to associate the content with personal interests and values and is more likely to opt for discussing the content with peers if the student experiences a personal connection with the subject. Research shows that if students can identify subject content as relevant, they are more likely to find assignments more meaningful, increasing both motivation, engagement, and achievements (Assor et al., 2002; Hulleman et al., 2010; Rosenzweig et al., 2020).

There are several studies within the SDT framework that highlights the importance of relevance and how it relates to promoting student motivation (see, e.g., Jang, 2008; Steingut et al., 2017; Vansteenkiste et al., 2018). Deci et al. (1994) conducted an experiment where students were to conduct a mundane task (pressing the space bar in front of a computer at random intervals whenever a dot appeared on screen), but a group of students were provided with a rationale (that performing this exercise increased concentration levels) and a noticeable effect on the quality of internalization was reported. In a similar experiment, Reeve et al. (2002) provided higher education Chinese language students with rationales (such as emphasizing that the learning activity was important since they themselves potentially could have their own Chinese language students one day) and found an increase in not only autonomous forms of motivation, but that the students also exerted more effort in the assignment. In a more recent study, Savard et al. (2013) conducted an experiment during a clinical workshop in interpersonal problem solving. The attendees were split into two groups with identical tasks, but where the experimental group were provided with extra instructions emphasizing how this assignment would translate to real-world scenarios. Not only did the experimental group experience more autonomous motivation, but they also experienced a decrease in negative emotions relative to the control group.

### Present study

The overarching aim of this study was to investigate the effect of introducing more relevant learning exercises on motivation and student functioning in a university statistics course. Although the assignments and exercises in this statistics course are relevant for the curriculum, students from other STEM backgrounds might perceive the assignments as less relevant from their perspectives given their disciplinary background. Most research within STEM education on personal relevance or meaning has primarily focused on enhancing perceived meaning among students by asking them to link the subject content to their own personal beliefs and goals (see, e.g., Gaspard et al., 2015; Harackiewicz et al., 2016; Harackiewicz and Priniski, 2018). Further, research on personal meaning has mainly been conducted on courses within biology and physics in higher educations (see, e.g., Hulleman et al., 2010; Tibbetts et al., 2016; Canning et al., 2018; Rosenzweig et al., 2020). Although results indicate a prominent effect on learning outcomes and motivation, it is important to expand the research to other branches within STEM educations. It may be easier to relate subject content to personal experiences and values in disciplines such as biology compared to more abstract fields like mathematics or statistics, where assignments and problems can appear more disconnected from everyday life. Hence, we are extending this research into numeracy disciplines in our study.

The variables of interest were the SDT motives (amotivation, external regulation, identified regulation, intrinsic motivation), vitality, effort, and emotional affect. We tested three hypotheses:

1) Does making course content more relevant to students increase perceived autonomous forms of motivation, vitality, and effort (H1)?
2) Does learning content perceived as more relevant by students increase their positive emotions and decrease their negative emotions (H2)?
3) Finally, we propose an extensive path model in which we hypothesized that autonomous forms of motivation predict effort, vitality, and negatively relate to negative affect, whereas controlled forms of motivation negatively predict effort, vitality, and positively predict negative affect (H3).

## Materials and methods

### Participants

The participants consisted of 67 (52.2% males) second-year undergraduate STEM students. To protect anonymity, age was asked in intervals (3.0% were 20–21 years, 97.0% were > 21 years). The students were recruited from an introductory statistics course for STEM students. Participation was voluntary, and no reward was offered. The students were informed about the project and the data were treated confidentially. The study obtained formal approval from the Norwegian Centre for Research Data (NSD).

### Procedure

During a mandatory computer exercise, students were randomly assigned into an experimental group or a control group based on which exercise set they received upon entering the classroom. Both exercises focused on the same statistical topics, were of similar length, and the students were asked to work individually. In the control condition, (i.e., the “generic” exercise set) the students were given data from a simulated game of darts and were tasked to determine which dart was better based on a set of score cards. They were supposed to use paired t-tests, t-tests with equal and non-equal variances, and determine normality graphically. In the experimental condition (i.e., the “relevant” exercise set), the students were provided with genuine climate data from a research center in Greenland that focused on ice smelting and global warming. The exercise was framed as a case where the students had to determine if temperature had increased over the last 50 years. The students were using the same statistical techniques as in the control group. Both groups used the statistical software (RStudio, 2023) for the exercise, and submitted a final report with the interpretation of the results after the exercise was complete. Prior to the experiment, students were given a short pre-test questionnaire measuring emotional affect. After the experiment, students were given a post-test questionnaire measuring emotional affect, motivation, vitality, and effort. The study was conducted at the end of the semester to minimize differences due to possible prior experience using RStudio between the students.

### Measures

#### Positive and negative affect

We used the positive and negative affects scale (PANAS) to measure affect (Watson et al., 1988). Students were asked to score their current mood before starting the exercise and again at the end of the exercise. Students responded on a seven-point Likert scale, ranging from 1 (not true at all) to 7 (very true). In previous studies, this scale has been shown to be both valid and reliable (Crawford and Henry, 2004). The Cronbach’s alpha was acceptable in both pre-test and post-test measures; pre-test positive affect (α = 0.95), pre-test negative affect (α = 0.95), post-test positive affect (α = 0.94) and post-test negative affect (α = 0.94).

#### Situational motivation

Situational motivation was measured using the 16-item Situational Motivation Scale (SIMS; Guay et al., 2000). The SIMS measures different regulations. The students were presented with the statement “Why are you currently doing this exercise?” and were given situational responses matching the different regulations. Item examples are “There may be good reasons to do this activity, but personally I do not see any” (amotivation), “Because I’m supposed to do it” (external regulation), “Because I’m doing it for my own good” (identified regulation), and “Because this activity is fun” (intrinsic motivation). The students were asked to rate the statement on a seven-point Likert scale, ranging from 1 (not true at all) to 7 (very true). Previous studies have found reliable results for this scale in higher education (Østerlie et al., 2019). The Cronbach’s alphas were acceptable for amotivation (α = 0.94), external regulation (α = 0.82), identified regulation (α = 0.81), and intrinsic motivation (α = 0.83).

#### Subjective vitality

The seven-item subjective vitality scale was used to measure student vitality (Ryan and Frederick, 1997). Previous studies have found reliable results for this scale in higher education (Bostic et al., 2000). Vitality was measured on a seven-point Likert scale, ranging from 1 (not true at all) to 7 (very true). An item example is “I feel alive and vital!.” The Cronbach’s alpha for the current study was found to be α = 0.96.

#### Effort

Effort was measured using the five-item Effort scale from the Intrinsic Motivation Inventory (Deci et al., 1994). The students were presented with statements such as “I put a lot of effort into this” and asked to rate this statement on a seven-point Likert scale, ranging from 1 (not true at all) to 7 (very true). This scale has proven to be reliable in previous research in higher education (Ostrow and Heffernan, 2018), and the Cronbach’s alpha for this scale was α = 0.94.

#### Value

Perceived value was measured using the sub-scale Value from the Intrinsic Motivation Inventory scale (Deci et al., 1994). The students were asked to score the scale on a seven-point Likert scale, ranging from 1 (not true at all) to 7 (very true). An item example from this scale is “I believe this activity could be of some value to me.” Previous studies have found reliable results for this scale in higher education (Tsigilis and Theodosiou, 2003). The Cronbach’s alpha was α = 0.92.

### Analytical strategy

All statistical analyzes were performed using the open-source program RStudio (RStudio, 2023). Descriptive statistics were assessed by means of the Lavaan package (Rosseel, 2012). To analyze hypothesis 1, we performed *t*-tests. The strength of the mean differences was assessed using Cohen’s d. For hypothesis 2 we conducted a Wilcoxon rank test to test for significant differences between the pre- and post-tests. Structural equation modeling was employed to investigate the path model for hypothesis 3. To evaluate the goodness-of-fit for the model, the indices SRMR, CFI, TLI, RMSEA, and *χ*^2^ were employed. The SRMR (Standardized Root Mean Square Residuals) is a measurement for the average absolute correlation-residuals, or the discrepancy between the observed and expected correlations. The CFI (Comparative Fit Index) is a goodness-of-fit statistic measuring the discrepancy from the model to the independent null-model. The RMSEA (Root Mean Square Error of Approximation) is a fit index that describes the discrepancy between the hypothesized model and a perfect model. The final fit index is the Chi-square goodness of fit test, which indicates how well a theoretical distribution from the model matches the actual empirical distribution. A good model fit is indicated by SRMR <0.08, CFI > 0.90, TLI > 0.95, and RMSEA <0.08. Finally, *χ*^2^ *p* > 0.05 is generally an indication of a good fit (Shi et al., 2018).

## Results

### Descriptive analyses

Out of the 67 students, two students (3.0%) did not complete the rest of the questionnaire after the initial pre-test questionnaire. Given the low missingness in the data (i.e., < 5%), we imputed the data (Rubin, 1987). Missing data were imputed using the Expectation–Maximization (EM) algorithm (Do and Batzoglou, 2008).

To ensure successful randomization, we included three manipulation checks. We performed two independent t-tests which indicated no significant differences between the control and experimental condition for students who worked alone compared to those who worked in collaboration with others (*p* > 0.05) and baseline knowledge of the R software (*p* > 0.05). Thus, our manipulation check indicated no bias regarding students working alone or in collaboration and baseline knowledge in R. To check for the success of the assignment manipulation (i.e., “interesting/relevant” exercise versus “generic/traditional” exercise), we measured interest (the scale was derived from the Intrinsic Motivation Inventory, Cronbach’s alpha was α = 0.97) as a proxy for relevance and compared the scores of the two groups to see if the subjects in the experimental condition found the assignments more relevant. Consequently, there was a significant difference (*p* < 0.05) between the groups (i.e., interest was higher in the experimental group). To test for any systematic gender differences in our sample, we conducted a range of independent t-test between gender in our study variables. Only one gender difference was found, suggesting that women scored higher on vitality compared to men (p < 0.05). Hence, we collapsed gender across all our study variables.

The study variables were all within a normal distribution except amotivation, value, and negative affect (pre- and post-test), which are somewhat skewed but within an acceptable range (Table 1). Correlation analyses show that intrinsic motivation and identified regulation are positively correlated with vitality and effort, whereas external regulation and amotivation are negatively correlated with the same variables.

**Table 1:** Descriptive statistics for main variables.

	*M*	*Range*	*SD*	*Skewn.*	*Kurt.*	*α*
Intrinsic motivation	4.38	1–7	1.29	0.04	−1.14	0.83
Identified regulation	4.79	1–7	1.10	−0.16	−0.89	0.81
External regulation	5.46	1–7	1.08	−0.64	−0.17	0.82
Amotivation	1.83	1–7	1.06	1.52	1.50	0.94
Subjective vitality	3.90	1–7	1.35	0.10	−1.11	0.96
Effort	4.91	1–7	1.39	−0.13	−1.35	0.94
Value	5.73	1–7	0.91	−0.93	1.03	0.92
Interest	3.79	1–7	1.66	−0.07	−1.57	0.97
Positive affect (pre-test)	4.00	1–7	1.07	−0.44	−0.66	0.95
Negative affect (pre-test)	1.42	1–7	0.56	0.85	−0.83	0.95
Positive affect (post-test)	3.57	1–7	0.99	−0.31	−0.69	0.94
Negative affect (post-test)	1.82	1–7	0.95	1.04	0.37	0.94

### Main analyses

#### How relevant assignments can affect autonomous motivation, effort, and vitality

**Figure 1:**
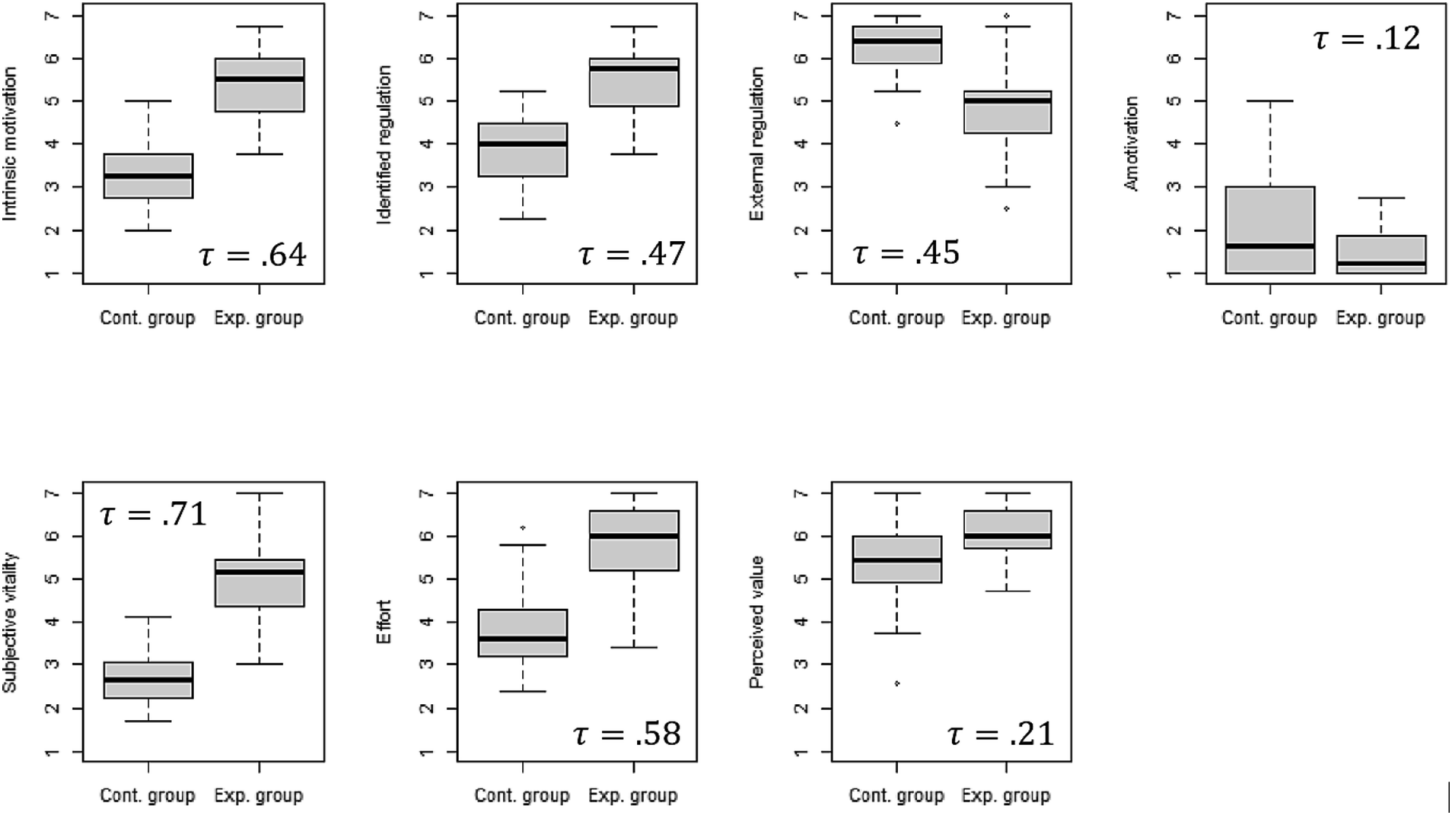
Box plots for the main variables. Plots depicting the differences between the study variables in the control group and the experimental group. τ denotes the proportion of explained variance for each dependent variable.

We used independent t-tests to investigate if more relevant exercises predict more autonomous forms of motivation (Hypothesis 1). Results show that students in the experimental condition reported higher intrinsic motivation, higher identified regulation, and higher vitality relative to the control group (Figure 1; Table 2). Students in the control condition reported higher external regulation and amotivation compared to the experimental condition. The strength of the mean difference between the conditions indicates a strong effect size for intrinsic motivation (*d* = 1.58), identified regulation (*d* = 1.36), external regulation (*d* = −1.34), subjective vitality (*d* = 1.67), effort (*d* = 1.51), and value (*d* = 0.92), and a moderate effect size for amotivation (d = −0.67).

**Table 2:** Mean differences and *t*-values for the main variables.

	*Mean difference*	*t*	*p*
Intrinsic motivation	2.03	−10.72	***
Identified regulation	1.51	−7.58	**
External regulation	−1.44	7.52	***
Amotivation	−0.72	2.82	**
Subjective vitality	2.24	−12.59	***
Effort	2.09	−9.33	***
Value	0.83	−4.01	**
Interest	2.50	−3.29	***

**p < 0.01, ***p < 0.001.

#### How relevant tasks can affect positive and negative emotions

**Figure 2:**
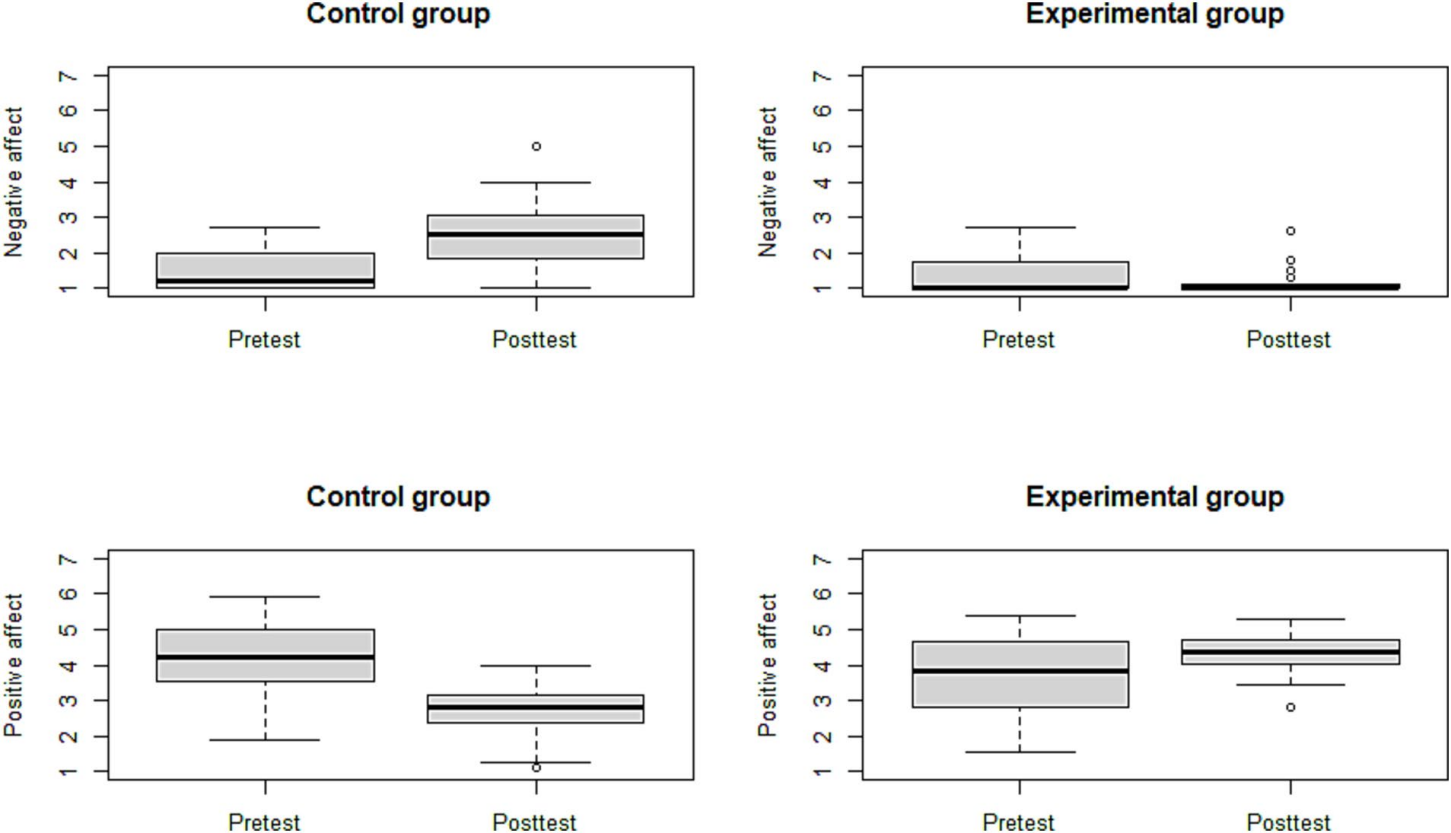
Box plots depicting the pre-test post-test results.

We conducted a Shapiro–Wilk normality test to determine normality due to the skewness of the negative affect measures. Since both the pre-test and post-test measures for negative affect failed the normality test (*p* < 0.001), a Wilcoxon rank test was employed to test for significant differences between the pre-test and post-test (Hypothesis 2; the experimental condition will enhance positive affect and reduce negative affect from pre- to post-test). Results indicate no significant differences in the pre-tests for either negative or positive affect between the experimental or control group. We did, however, find a significant difference between the pre-test and post-test for the control group for both positive and negative affect, but we found no significant difference between the pre-test and post-test for the experimental condition (see Figure 2). However, there were significant differences between the post-tests for the control group and the experimental group, both for positive and negative affect, indicating that students in the control group reported higher negative affect than the experimental condition, and lower positive affect than the experimental condition.

#### The relationship between value, motivation, effort, and vitality

To test Hypothesis 3, we used path analysis to investigate the multivariate relations between our study variables. We specified a process model in which condition (control vs. experiment) would predict value which in turn predict the different regulations, which in turn predict vitality, negative affect, and effort. We cannot infer causality as these data were cross-sectional in nature. However, model directionality is based on strong theoretical SDT propositions and previous research (see, e.g., Assor et al., 2002; Weber, 2003; Burton et al., 2006; Jang, 2008; León et al., 2015; Núñez and León, 2016; Monteiro et al., 2018; Vansteenkiste et al., 2018; Feng et al., 2019; Waterschoot et al., 2019), thus this design is considered appropriate for the purpose of this study (Bollen and Pearl, 2013).

**Figure 3:**
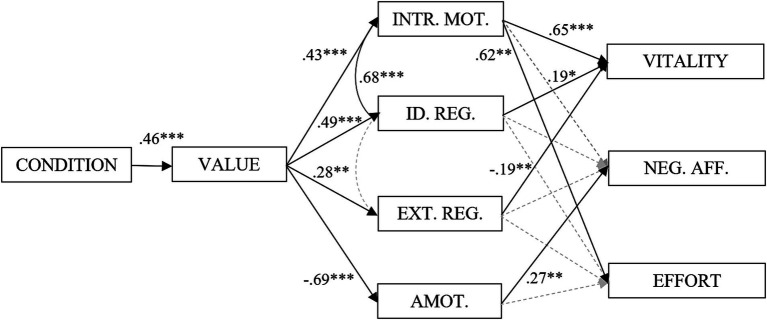
Modified pathway diagram with standardized regression coefficients. Figure of the path-model with standardized regression coefficients. Dotted lines indicate non-significant path coefficients. *p < 0.05, **p < 0.01, ***p < 0.001. NEG.AFF, Negative affect; INTR. MOT., Intrinsic motivation; ID. REG., Identified regulation; EXT. REG., External regulation; AMOT., Amotivation. CONDITION (generic exercise coded = 0, relevant/interesting exercise coded = 1). Negative affect is standardized residual change scores.

As some of the variables highly correlate (Table 3), the potential presence of multicollinearity must be addressed. However, calculations of the variance inflation factor (VIF) for all the variables indicate no redundant variables, VIF < 10 (Kline, 2016). Model fit for our baseline model indicated a poor model-fit, with *p*(*χ*^2^ = 5854.24, df = 253) = 0, CFI = 0.67, TLI =0.26, RMSEA = 0.42, and SRMR = 0.24. We employed a modification index algorithm (Jorgensen, 2017) to our model indicating that intrinsic motivation should covary with identified regulation, and that identified regulation should covary with external regulation. Our re-specified model (Figure 3) indicates acceptable model fit, p(χ^2^ = 5674.24, df = 248) = 0.054, CFI = 0.987, TLI = 0.942, RMSEA = 0.117 (CI: 0.000, 0.205), and SRMR = 0.035. Using a chi-square difference test to assess if the re-specified model was significantly different than the baseline model, we found a significant difference between the baseline and the modified model (*p* < 0.01). Specifically, results (Figure 3) indicate that the experimental condition predicts value. In turn, value predicts intrinsic motivation, identified regulation, and external regulation, and is negatively related to amotivation. Intrinsic motivation predicts vitality and effort, whereas identified regulation predicts vitality but not effort. Finally, external regulation is negatively related to vitality while amotivation predicts negative affect.

**Table 3:** Correlation matrix of the main variables.

	1	2	3	4	5	6	7	8	9
1. Intrinsic motivation	–								
2. Identified regulation	0.90***	–							
3. External regulation	−0.41***	−0.25**	–						
4. Amotivation	−0.60***	−0.60***	−0.03	–					
5. Vitality	0.88***	0.81***	−0.51***	−0.46***	–				
6. Effort	0.78***	0.68***	−0.45***	−0.34**	0.80***	–			
7. Value	0.71***	0.70***	−0.09	−0.70***	0.60***	0.49***	–		
8. Negative affect†	−0.62***	−0.50***	0.53***	0.41***	−0.70***	−0.64***	−0.38**	–	
9. Positive affect†	0.82***	0.79***	−0.60***	−0.35**	0.78***	0.76***	0.52***	−0.54***	–

*p < 0.05, **p < 0.01, ***p < 0.001. Variables marked † are composed from residuals from the pre- and post-tests.

## Discussion

The main aim of this study was to investigate how making a statistics exercise more relevant for students can impact motivation, well-being, effort, and affect. Results indicate that providing a more relevant exercise had a strong effect on autonomous motivation, effort, and vitality in students. These results are in line with previous findings in similar studies (see, e.g., Diseth et al., 2017; Cheon et al., 2018). By providing a rationale in exercises and assignments, one can help students understand why the learning material can have meaningful utility, thus fostering more autonomous regulations (Deci et al., 1994). When students act and engage with the learning content out of their own volition, they are acting true to themselves and their interests. Controlling behavior can feel energy depleting as there is an internal conflict with the external pressure that forces the behavior, hence autonomously motivated students are more prone to experiences of feeling energized with a surplus of energy (Levesque et al., 2004; Mouratidis et al., 2011; Vansteenkiste et al., 2018). This is in line with our findings, where autonomously motivated students reported higher levels of vitality. This result is similar to previous studies by for instance Nix et al. (1999), Martela et al. (2016), and Tsoi et al. (2018). Further, we found that the students in the experimental group exerted more effort into the learning task, a result supported by earlier research (see, e.g., Kusurkar et al., 2013; Kusurkar and Croiset, 2015). When students are more autonomously motivated, the reason for their behavior is now inherent to the learning activity itself, and students will naturally gravitate toward the content and willingly put more effort into the activities (Vansteenkiste et al., 2018). Effort is an important constituent in learning as it is associated with academic achievements, increased recollection, and perceived competence, hence it constitutes a wide aspect of student functioning traits (Schmid and Bogner, 2015; Xu et al., 2018; Jiang et al., 2019).

We found that students’ positive and negative affect changed during the exercise, partly in line with our hypothesis. Within the control group, negative affect increased, and positive affect decreased during the exercise. This supports a previous finding by Joussemet et al. (2004), where children who experienced controlled motivation reported lower positive affect relative to an experimental group. Whenever students are extrinsically regulated, their motivation will take a more controlling form due to the behavior being controlled by some external pressure such as seeking approval or avoiding punishment and is often associated with lower well-being and negative emotions as the learning activity is perceived as less enjoyable (Pelletier et al., 2001; Reeve et al., 2002; Cerasoli et al., 2014; Howard et al., 2021). Contrary to our hypothesis, we found no difference in neither positive nor negative affect for the experimental group. These results contradict a study by Kocayoruk (2012) who reported that both positive and negative affect were related to autonomy. Basu and Bano (2016) found that intrinsic motivation correlated negatively with negative affect, but also that controlled motivation correlated positively with negative affect, and no significant correlation was found between controlled motivation and positive affect. A possible explanation for this inconsistency regarding affect could lie in distinguishing the different emotions based on valence. For instance, when feeling anger, one often attributes negativity toward others, while sadness is associated with feeling alone or helplessness (Frijda et al., 1989). Failing to distinguish how these negative emotions manifest in the various settings could induce different global results.

The process model generally supported the hypothesis (3) that condition (i.e., relevant/interesting exercise vs. generic/traditional exercise) predicts value which in turn predicts autonomous forms of motivation, extrinsic regulation and amotivation. When learning content is connected or related to a student’s own interests instead of being some arbitrary construct, i.e., more relevant and in line with the student’s own personal values, the learning content is instead perceived as meaningful from their perspective (Canning and Harackiewicz, 2015). If the learning content has meaningful merit to the students, it is more likely that a better internalization occurs, thus the students are more likely to be identified regulated instead of experiencing a controlling contingency to drive behaviors (Hulleman et al., 2010). Our findings are in line with previous research (see, e.g., Katz et al., 2006), although there are some discrepancies in our model compared to the data. We added a covariance between intrinsic motivation and identified regulation as well as a covariance between identified regulation and external regulation according to the modification index algorithm in our final model, a correction that is supported in SDT studies (see, e.g., Wang and Guthrie, 2004). The magnitude of the covariance is similar to previous studies (Ketonen et al., 2018). Further, we found that autonomous forms of motivation predict vitality. This is supported by previous research (see, e.g., Gillet et al., 2013; Lemos et al., 2017; Tsoi et al., 2018). Further, in line with previous research (see, e.g., Gillet et al., 2013), we found that amotivation predicts negative affect, while intrinsic motivation positively predicts vitality. Since amotivation is associated with a relative lack of motivation in general, performing an action while amotivated does come with the cost of negative emotions (Gillet et al., 2013). These findings have also been reported in a study by Núñez and León (2016). We did, however, not find any relationship between identified regulation and effort, contrary to what Liang et al. (2018) reported in a similar study.

## Limitations

There are several limitations worth mentioning when interpreting the results of our study. First, this study highlights the importance of providing students with relevant and interesting tasks to foster autonomous motivation, and the results in this study generally support previous research conducted within the realm of SDT. Given that this was a classroom experiment, our sample size was limited by the number of students that enrolled in the course. The course was mandatory for undergraduate students from different disciplines, including biology, geology, and oceanography. We recommend future studies to replicate our study with more heterogenous student samples, and a larger sample size.

Second, we aimed at creating a relevant/interesting exercise, and in doing so provided a discipline relevant case with a contemporary narrative. Since we only investigated a single exercise, we cannot conclude if the findings are due to the disciplinal content in the exercise. Future studies need to investigate if other exercises based on other cases have a similar impact on students’ motivation and well-being. Students from various disciplines could have different experiences if the content in the exercises were tailored toward their specific fields which could have been controlled by tailoring different exercises to each STEM discipline.

Another limitation arises from the absence of controlling for the possible impact of the structural differences in approaching the problems between the two exercises. While the generic exercises in the control group were presented with punctual instructions on how to solve every problem, the students in the experimental group were only presented with the problems and then given more freedom on how to solve the exercises and were given very simple hints on possible R codes they could implement to solve the problems. Future research is encouraged to implement a design in which the autonomous narratives of the two exercises are more similar.

Next, the sample size could raise concerns regarding the use of path-analysis. SEM usually requires large samples to achieve adequate model fits. Yet, no conventional requirements exist, where suggested sample sizes vary from *N*/*q* = 5 to *N*/*q* = 20 (Kline, 2016). A problem using path modeling with small sample sizes arises from non-convergence of the model if the algorithm trying to determine model parameters that maximize the data likelihood cannot find a solution to satisfy the convergence criteria (Rosseel, 2020). In our study however, the model converged. Further, we found no negative variances or out-of-range parameters which is considered a positive sign of no structural model misspecification (Savalei, 2019). A third concern could lie in the parameter estimation at low sample sizes as the confidence intervals of the point estimates can be too narrow. However, simulations have shown (see, e.g., Wolf et al., 2013; Savalei, 2019) that variables can be reliably estimated even at sample sizes of *N* = 30 in path modeling given that the other aforementioned criteria are not violated.

Another limitation of the experiment could lie in the students’ valence of already being familiar with the phenomenon of the learning activity (i.e., familiarity with global warming). Future studies should replicate our experiment while controlling for the novelty factor of the learning content.

Further, the study utilized self-report measures. That is, instead of objective measures like grades or test scores, students’ self-reported perceived experiences were measured. Although research has shown that self-reported measures are reliable (Benton et al., 2013), they are susceptible to memory bias when the measurements become comprehensive (Pekrun, 2020). Further, self-reported measures have been shown to be less reliable among weak students (Kuncel et al., 2005).

A final limitation is that our study is based on manipulation of an exercise during one classroom activity. A longitudinal design with multiple exercises throughout the semester could potentially shed more light on causality and the dynamics of internalization on students’ well-being and motivation across a semester.

## Conclusion

In sum, the results indicate that making exercises and assignments more relevant for higher education students plays a pivotal role in pathing growth for psychological well-being, student functioning, and motivation in the classroom. Our intervention displayed positive effects on quality of motivation, vitality, affect, and effort among the students. Results indicate that making assignments more relevant for students is a strategy that can support healthier internalization of learning activities, and these findings provide important insight into how interventions could be utilized in higher education statistics courses to increase student motivation. Whereas similar studies have investigated the effect on internalization of making assignments more relevant, they are limited to less abstract STEM disciplines such as biology and physics, and hence our study is an important contribution since we are expanding the research into less tangible numeracy disciplines. We encourage future researchers to replicate and expand our current study by implementing interventions with different STEM contexts in assignments and exercises.

## Data availability statement

The raw data supporting the conclusions of this article will be made available by the authors, without undue reservation.

## Ethics statement

The studies involving humans were approved by Norsk senter for forskningsdata (NSD). The studies were conducted in accordance with the local legislation and institutional requirements. The participants provided their written informed consent to participate in this study.

## Author contributions

MJ: conceptualization, methodology, formal analysis, software, writing – original draft, and investigation. LJ: conceptualization, methodology, writing – review and editing, and supervision. SE: conceptualization, writing – review and editing, and supervision. All authors contributed to the article and approved the submitted version.

## Conflict of interest

The authors declare that the research was conducted in the absence of any commercial or financial relationships that could be construed as a potential conflict of interest.

## Publisher’s note

All claims expressed in this article are solely those of the authors and do not necessarily represent those of their affiliated organizations, or those of the publisher, the editors and the reviewers. Any product that may be evaluated in this article, or claim that may be made by its manufacturer, is not guaranteed or endorsed by the publisher.
